# Comparison  of  efficacy, safety, and costs between neoadjuvant hypofractionated radiotherapy and conventionally fractionated radiotherapy for esophageal carcinoma

**DOI:** 10.1002/cam4.2250

**Published:** 2019-05-22

**Authors:** Jiahua Lyu, Tao Liu, Tao Li, Fang Li, Qifeng Wang, Jie Wang, Yongtao Han, Junchao Wang, Jun Zhang, Lin Peng, Jinyi Lang

**Affiliations:** ^1^ Sichuan Cancer Hospital and Institute Chengdu China; ^2^ School of Medicine University of Electronic Science and Technology of China Chengdu China; ^3^ Ya'an people's hospital Ya'an China

**Keywords:** conventionally fractionated radiotherapy, costs, esophageal cancer, hypofractionated radiotherapy, neoadjuvant, prognosis

## Abstract

**Background:**

We compared the efficacy, safety, and costs of hypofractionated radiotherapy (HFRT) and conventional fractionated radiotherapy (CFRT) for the neoadjuvant treatment of esophageal cancer.

**Materials and Methods:**

Overall, 110 patients with esophageal cancer treated with neoadjuvant chemoradiotherapy from October 2002 to July 2017 were retrospectively included and divided into a HFRT group (42 patients received 30 Gray [Gy]/10 fractions for 2 weeks) and a CFRT group [68 patients received 40 Gy/20 fractions for 4 weeks]. Concurrent chemotherapy comprised cisplatin combined with either 5‐FU or taxane. Surgery was performed 3‐8 weeks after radiotherapy. We compared the outcomes, adverse events, and costs between the two groups.

**Results:**

Pathological downstaging was achieved in 78.6% of the HFRT group and 83.8% of the CFRT group (*P* = 0.612). Compared with the CFRT group, the HFRT group had similar pathological complete response (pCR) (33.3% vs 35.3%; *P* = 0.834), median overall survival (OS) (40.8 months vs 44.9 months; *P* = 0.772) and progression free survival (32.7 months vs 35.4 months; *P* = 0.785). The perioperative complication rates were also similar between the groups, but the treatment time and costs were significantly reduced in the HFRT group (*P* < 0.05). Finally, multivariate analysis identified cN0 stage, pathological downstaging and pCR as independent predictors of better OS.

**Conclusion:**

Preoperative HFRT is effective and safe for esophageal cancer. Moreover, it is similar to CFRT in terms of overall survival and toxicity and is cost effective and less time consuming.

## 1 INTRODUCTION

Neoadjuvant chemoradiotherapy has become a promising treatment for patients with stage II or III esophageal cancer. Several studies have reported that its use has improved local control, progression free survival (PFS), and overall survival (OS) in comparison with surgery alone.^1^, ^2^, ^3^ Conventional fractionated radiotherapy (CFRT) is the most commonly used neoadjuvant option for esophageal cancer, but the efficacy and safety of preoperative hypofractionated radiotherapy (HFRT), which delivers a dose larger than 2 Gray (Gy) per fraction in a lower overall dose, has also been studied in some patients with esophageal cancer.^4^, ^5^ These studies have indicated that preoperative HFRT could improve the local control rate of esophageal cancer and potentially increase patient survival compared with surgery alone. However, existing research has only compared surgery alone with preoperative HFRT plus surgery. To the best of our knowledge, there has been no comparison of HFRT and CFRT for the neoadjuvant treatment of esophageal cancer, and the optimal dose‐fractionation schedule remains undefined. Therefore, this retrospective study aimed to investigate and compare the efficacy, safety, and costs of neoadjuvant HFRT and the standard CFRT regimen for esophageal cancer.

## 2 MATERIALS AND METHODS

### 2.1 Patients

We retrospectively reviewed 110 patients with esophageal cancer who were treated with neoadjuvant chemoradiotherapy followed by surgery at authors^,^ institute from October 2002 to July 2017. Inclusion criteria were: (a) histologically or cytologically confirmed thoracic esophageal squamous cell carcinoma; (b) clinically stage II or III, as determined by the American Joint Committee on Cancer TNM 2002 staging system (version6.0)^6^; (c) Karnofsky performance status ≥80; (d) patients underwent neoadjuvant chemoradiotherapy followed by esophagectomy. Exclusion criteria were: (a) patients who had received previous anti‐tumor treatment for esophageal cancer before neoadjuvant chemoradiotherapy; (b) patients who had insufficient follow‐up data; (c) patients who had previous malignancy or other concomitant malignant diseases. Current study was undertaken in accordance with the ethical standards of the World Medical Association Declaration of Helsinki. A waiver of informed consent was requested, and this study was approved by authors^,^ institute.

Patients were divided into two treatment subgroups based on dose‐fractionation schedule of preoperative radiotherapy: (a) HFRT group; (b) CFRT group.

### 2.2 Preoperative treatment

All patients received preoperative intensity‐modulated radiation therapy concurrent with chemotharpy. In the HFRT group, patients received a total dose of 30 Gy in 10 fractions over 2 weeks, five fractions per week. In the CFRT group, patients received a total dose of 40 Gy in 20 fractions over 4 weeks, five fractions per week. The radiotherapy was delivered with photon energies of 6‐10 MV. The gross tumor volume (GTV) was defined by the primary tumor and any positive regional lymph nodes, which were determined using all available information, including physical examination, endoscopy, endoscopic ultrasonography, and neck‐thorax‐abdomen CT. The clinical target volume (CTV) provided a 3 cm margin in the proximal and distal direction and a 0.5 cm‐1.0 cm radial margin around the GTV. The planning target volume was defined by including an additional 0.5 cm margin of the CTV for tumor motion and set‐up variations. Neoadjuvant chemotherapy was concomitantly administered in all patients at 3‐week intervals (2 cycles) and comprised either cisplatin with 5‐fluorouracil (PF) or cisplatin with taxane (TP).

### 2.3 Surgery

Surgery was performed 3‐8 weeks after completion of radiotherapy. Esophagectomy and standard regional lymph node dissection, including two‐field or three‐field dissection, was performed. A group of pathologists examined the entire specimen with primary and dissected lymph nodes and reported the tumor type and extension, proximal and distal resection margins and lymph node status. Pathological downstaging was defined as a reduction in the clinical T or N stage compared with the pathological T or N stage prior to the start of neoadjuvant treatment. Pathologic complete response (pCR) was defined as no evidence of residual tumor cells in the primary site as well as in the resected regional lymph nodes.

### 2.4 Outcomes

Overall survival was calculated from the date of initial diagnosis to the date of death or last follow‐up. The time interval between initial diagnosis and local, regional recurrence, distant metastasis, death from any cause or last follow‐up without recurrence and metastasis was defined as PFS. The toxicity was evaluated according to the National Cancer Institute Common Terminology Criteria for Adverse Events (version 3.0). The radiotherapy and neoadjuvant treatment related time and costs were compared. The radiotherapy costs was calculated from the date of the radiation planning scan to the last radiotherapy treatment and included all associated radiation procedures including radiotherapy simulation, treatment plan generation, radiotherapy treatment, and image guidance. The total costs consisted of preoperative radiotherapy, chemotherapy, hospital stay charges, professional fees, imaging, all ensuing salvage and symptomatic supportive therapies, as incurred.

### 2.5 Statistical analyses

Statistical analyses were performed using SPSS 20.0 (SPSS Inc, Chicago, IL, USA). We used the Kaplan‐Meier method to estimate OS and PFS, with the log‐rank test to ascertain significance. The categorical variables between groups were compared using Pearson's Chi square test or Fisher's exact test, if indicated. Univariate and multivariate analysis with the Cox proportional hazards model was used to investigate the effect of different factors on survival. Covariates included treatment group (CFRT vs HFRT), sex, age (≤60 years vs >60 years), tumor location, KPS score (80 vs ≥90), clinical T‐stage (T1‐2 vs T3 vs T4), clinical N‐stage, chemotherapy regimens, pCR, and pathological downstaging. A two‐tailed *P*‐value less than 0.05 was considered statistically significant.

## 3 RESULTS

### 3.1 Patient characteristics

The clinical characteristics of the participants in the HFRT group (n = 42) and the CFRT group (n = 68) are summarized in Table 1. There were no significant differences in any clinical features between the two treatment groups.

**Table 1:** Baseline characteristics for patients in HFRT group vs CFRT group

Characteristics	HFRT group (n = 42)	CFRT group (n = 68)	*P*
Sex			0.454
Male	32	57	
Female	10	11	
Age, years			0.539
≤60	29	42	
>60	13	26	
Tumor location			0.960
Upper thoracic	9	14	
Middle thoracic	25	43	
Lower thoracic	8	11	
KPS score			1.000
≥90	25	40	
80	17	28	
cT‐stage^a^			0.732
T1‐2	3	8	
T3	27	41	
T4	12	19	
cN‐stage^a^			0.426
N0	19	25	
N1	23	43	
Chemotherapy regimens			
TP	12	27	0.306
PF	30	41	

Abbreviations: CFRT, conventional fractionated radiotherapy; HFRT, hypofractionated radiotherapy; KPS, Karnofsky performance status; PF, cisplatin with 5‐fluorouracil; TP, cisplatin with taxane.

a AJCC 2002 staging system.

### 3.2 Pathology

In the HFRT group, 40 patients (95.2%) underwent R0 resection compared with 64 (94.1%) in the CFRT group (*P* = 1.000). pCR was achieved in 14 patients (33.3%) after HFRT and 24 patients (35.3%) after CFRT (*P* = 0.834). Regarding the distribution of pathologic stages, patients in the HFRT group underwent similar downstaging compared with the CFRT group (78.6% vs 83.8%, *P* = 0.612) (Table 2).

**Table 2:** Distribution of pathologic stage groups after surgery

Pathologic stage group	HFRT group (n = 42)	CFRT group (n = 68)	*P*
pT‐stage			0.338
T0	16	30	
T1	4	10	
T2	12	9	
T3	9	18	
T4	1	1	
pN‐stage			1.000
N0	25	41	
N1	17	27	
pCR	14 (33.3%)	24 (35.3%)	0.834
Pathologic downstaging	33 (78.6%)	57 (83.8%)	0.612

Abbreviations: CFRT, conventional fractionated radiotherapy; HFRT, hypofractionated radiotherapy; pCR, pathologic complete response.

### 3.3 Survival

**Figure 1:**
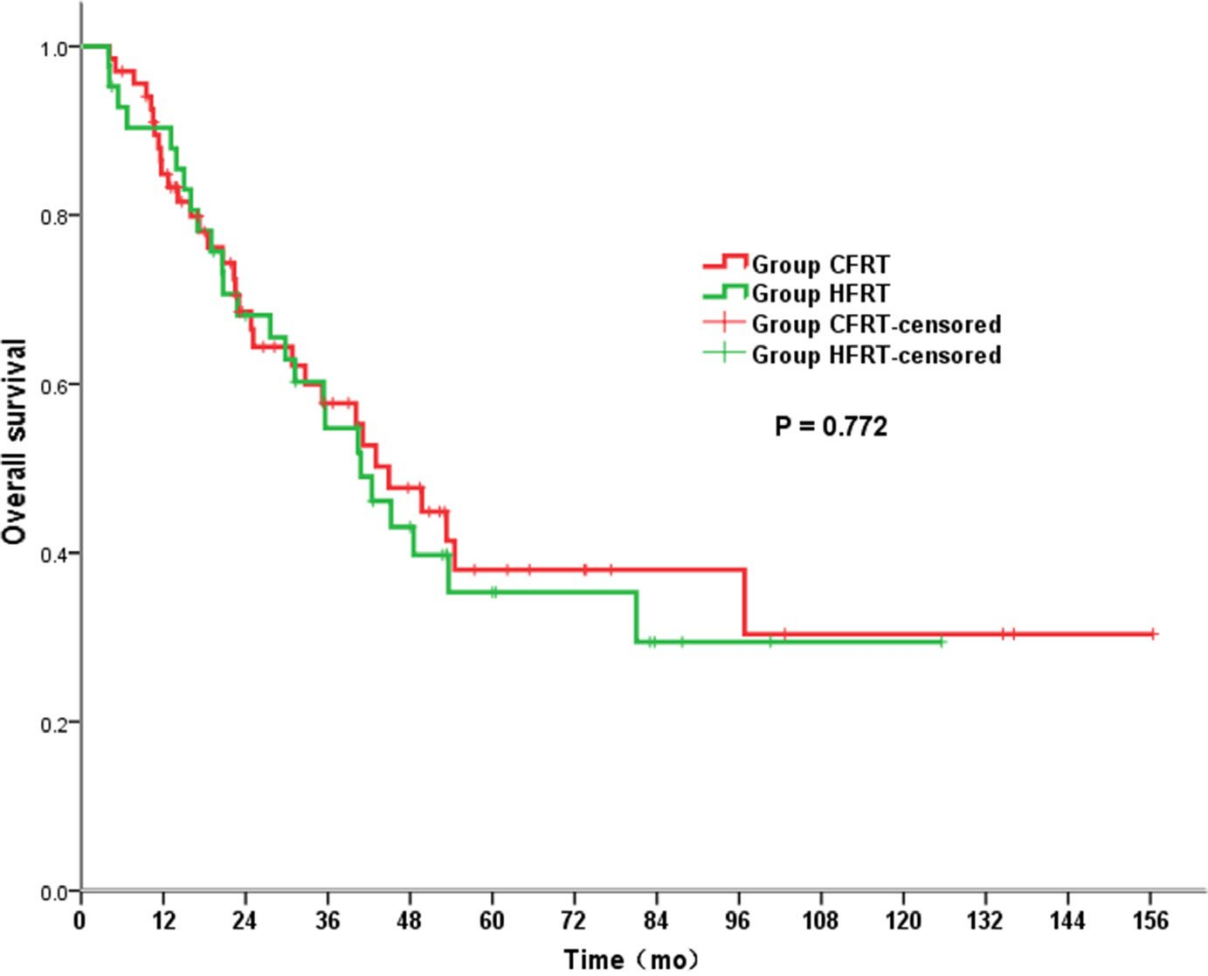
Overall survival for patients in HFRT group vs CFRT group. CRFT, conventionally fractionated radiotherapy; HFRT, hypofractionated radiotherapy

**Figure 2:**
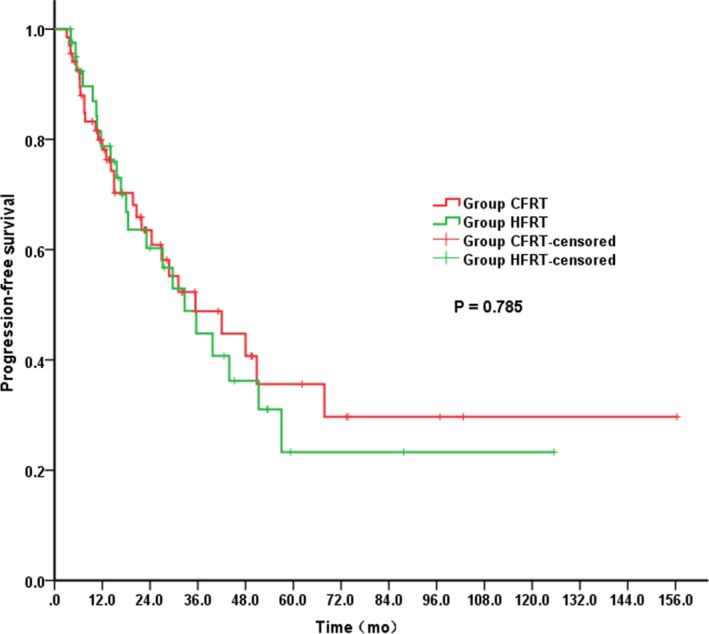
Progression‐free survival for patients in HFRT group vs CFRT group. CRFT, conventionally fractionated radiotherapy; HFRT, hypofractionated radiotherapy

The median follow‐up across the whole study population was 33 months. Kaplan‐Meier analysis for OS and PFS revealed no significant difference between the groups. The median OS was 40.8 and 44.9 months in the HFRT and CFRT groups, respectively (*P* = 0.772; Figure 1). More specifically, the respective OS rates in the HFRT and CFRT group were 90.4% and 84.9% at 1 year, 54.8% and 57.7% at 3 years and 35.3% and 38.0% at 5 years. The median PFS was 32.7 months in the HFRT group compared with 35.4 months in the CFRT group (*P* = 0.785; Figure 2). As summarized in Table 3, multivariate analysis indicated that clinical N0 stage (*P* = 0.018), downstaging (*P* = 0.020) and pCR (*P* = 0.039) were independent predictors of a better OS (Table 3).

**Table 3:** Univariate and multivariate analysis demonstrating factors associated with OS

Subgroup	Univariate analysis		Multivariate analysis	
	HR (95% CI)	*P*	HR (95% CI)	*P*
Treatment group				
CRFT	1.00		1.00	
HFRT	1.080 (0.639‐1.826)	0.772	1.062 (0.609‐1.850)	0.833
Sex				
Male	1.00		1.00	
Female	0.871(0.451‐1.682)	0.680	0.541 (0.256‐1.144)	0.108
Age, years				
≤60	1.00		1.00	
>60	0.814(0.456‐1.452)	0.486	0.982 (0.509‐1.897)	0.958
Tumor location				
Upper thoracic	1.00		1.00	
Middle thoracic	0.730 (0.394‐1.350)	0.315	0.567 (0.284‐1.129)	0.106
Lower thoracic	0.924 (0.414‐2.063)	0.847	0.559 (0.222‐1.405)	0.216
KPS score				
80	1.00		1.00	
≥90	0.787 (0.456‐1.356)	0.388	0.799 (0.443‐1.439)	0.455
Clinical T‐stage				
T1‐2	1.00			
T3	2.443 (0.747‐7.991)	0.139	1.795 (0.923‐13.056)	0.356
T4	3.479 (1.010‐11.989)	0.048	3.471 (1.133‐3.799)	0.066
Clinical N‐stage				
N0	1.00		1.00	
N1	1.794 (1.028‐3.130)	0.040	2.075 (1.133‐3.799)	0.018
Chemotherapy regimens				
PF	1.00			
TP	0.859 (0.475‐1.553)	0.614	0.806 (0.429‐1.515)	0.503
pCR				
Yes	1.00		1.00	
No	2.483 (1.359‐4.535)	0.003	2.012 (1.036‐3.908)	0.039
Downstaging				
Yes	1.00		1.00	
No	2.810 (1.471‐5.370)	0.002	2.332 (1.145‐4.752)	0.020

Abbreviations: CRFT, conventionally fractionated radiotherapy; HFRT, hypofractionated radiotherapy; KPS, Karnofsky performance status; pCR, pathologic complete response; PF, cisplatin with 5‐fluorouracil; TP, cisplatin with taxane.

### 3.4 Toxicities

Table 4 summarizes the haematological and non‐haematological toxicities observed during neoadjuvant chemoradiotherapy. Leukopenia was the most common grade 3 or 4 adverse event. Overall, 13 of the 42 patients who received HFRT (31.0%) developed grade 3 or 4 leukopenia compared to 25 of the 68 patients who received CFRT (36.8%) (*P* = 0.546). However, neither the adverse events during chemoradiotherapy nor the postoperative complications differed significantly between the two groups (Table 4). No deaths occurred within 30 days after surgery in either group.

**Table 4:** Adverse events during chemoradiotherapy and postoperative complications

	HFRT group (n = 42)	CFRT group (n = 68)	*P*
Adverse events during chemoradiotherapy			
Leukopenia			
Any grade	36 (85.7%)	61 (89.7%)	0.555
≥Grade 3	13 (31.0%)	25 (36.8%)	0.546
Anemia			
Any grade	22 (52.4%)	38 (55.9%)	0.844
≥Grade 3	2 (4.8%)	5 (7.4%)	0.706
Thrombocytopenia			
Any grade	12 (28.6%)	16 (23.5%)	0.653
≥Grade 3	2 (4.8%)	3 (4.4%)	1.000
Anorexia/Vomiting			
Any grade	19 (45.2%)	33 (48.5%)	0.845
≥Grade 3	1 (2.4%)	3 (4.4%)	1.000
Radiation esophagitis			
Any Grade	16 (38.1%)	22 (32.4%)	0.680
≥Grade 3	2 (4.8%)	2 (2.9%)	1.000
Radiation pneumonitis			
Any grade	6 (14.3%)	13 (19.1%)	0.609
≥Grade 3	0 (%)	1 (1.5%)	1.000
Postoperative complications			
Anastomotic leakage	5 (11.9%)	5 (7.4%)	0.501
Hemorrhage	1 (2.4%)	0 (0.0%)	0.385
Chylothorax	1 (2.4%)	1 (1.5%)	1.000
Pulmonary infection	5 (11.9%)	12 (17.6%)	0.588
Pleural effusion	4 (9.5%)	9 (13.2%)	0.778
Incision infection	2 (4.8%)	2 (2.9%)	0.635
Arrhythmia	2 (4.8%)	5 (7.4%)	0.706
Injury of recurrent nerve	1 (2.4%)	1 (1.5%)	1.000

Abbreviations: CRFT, conventionally fractionated radiotherapy; HFRT, hypofractionated radiotherapy.

### 3.5 Treatment time and cost analysis

The median times for preoperative treatment were 69.00 ± 13.50 and 80.46 ± 11.74 days in the HFRT and the CFRT groups, respectively (*P* = 0.000). It took a median of 16.71 ± 4.22 days to deliver neoadjuvant radiotherapy in the HFRT group compared with 32.85 ± 5.09 days in the CFRT group (*P* = 0.000). (Table 5).

**Table 5:** Treatment time and cost analysis

	HFRT group (n = 42)	CFRT group (n = 68)	*P*
Radiotherapy time, days	16.71 ± 4.22	32.85 ± 5.09	0.000
Preoperative treatment time, days	69.00 ± 13.50	80.46 ± 11.74	0.000
Radiotherapy cost, yuan	14218.67 ± 5424.12	28750.94 ± 7093.25	0.000
Preoperative total cost, yuan	23205.86 ± 5862.65	39170.38 ± 8752.78	0.000

Abbreviations: CRFT, conventionally fractionated radiotherapy; HFRT, hypofractionated radiotherapy.

Table 5 summarizes the average total and radiotherapy‐related costs for each group during neoadjuvant treatment. The average total costs for patients treated with HFRT were significantly less (23205.86 ± 5862.65 yuan) compared with that for those treated with CFRT (39170.38 ± 8752.78 yuan; *P* = 0.000). The average costs related to radiotherapy were also significantly lower in the HFRT group (14218.67 ± 5424.12 yuan) than in the CFRT group (28750.94 ± 7093.25 yuan; *P* = 0.000).

## 4 DISCUSSION

In recent years, the need for neoadjuvant chemoradiotherapy has increasingly been recognized in esophageal cancer. There are multiple advantages to this therapy. First, the local blood supply and oxygenation of a tumor is important for drug delivery in chemotherapy and sensitivity in radiotherapy, so these procedures are best performed when tumor blood vessels are not damaged by surgery. Second, neoadjuvant chemoradiotherapy can downstage tumors, improving their resectability and decreasing the rate of positive surgical margins. Third, the necrosis and fibrosis of tumor tissue after neoadjuvant chemoradiotherapy greatly reduces active tumor cells and the probability of tumor cells shedding, spreading, and planting during surgery, thereby reducing local recurrence rates. Compared with esophagectomy alone, several randomized studies have indicated that this neoadjuvant chemoradiotherapy improves pCR, locoregional control, and survival in patients with esophageal cancer undergoing esophagectomy.^1^, ^2^, ^7^

Conventional fractionated radiotherapy has become the mainstay of preoperative radiotherapy in esophageal cancer. However, the extension of preoperative treatment time and the increase of treatment cost is an issue that must be considered. How can we reduce the treatment time and costs while maintaining the efficacy and safety? HFRT may provide us with a new strategy. It was postulated that a HFRT schedule that delivers doses larger than 2 Gy per fraction, but with a lower overall dose, improves dose escalation strategies to increase tumor control and helps maintain dose equivalence for tumor cure while decreasing the dose delivered to normal tissue.^8^, ^9^ There have been many studies showing the benefits of HFRT in lung cancer,^10^, ^11^ prostate cancer,^12^, ^13^ breast cancer,^14^, ^15^ advanced head and neck cancer 16, 17 and locally advanced inoperative esophageal cancer.^18^, ^19^ Regarding preoperative HFRT, colorectal cancer is the most studied cancer, with research indicating that preoperative HFRT (also called short‐course radiotherapy) is as effective as conventional radiotherapy (also called long‐course radiotherapy) in terms of long‐term survival.^20^, ^21^, ^22^

Prior to our study, only few studies focused on the use of preoperative HFRT followed by surgery in esophageal cancer. Walsh et al^4^ conducted a prospective, randomised trial comparing surgery alone and a combination of HFRT, chemotherapy and surgery. Patients assigned to the multimodal therapy received two courses of chemotherapy and a course of radiotherapy (40 Gy/15 fractions over 3 weeks), followed by surgery, and it was reported that pCR was achieved in 25% of patients who underwent multimodal therapy. The median OS among patients assigned to receive multimodal therapy was 16 months, compared with 11 months for those assigned to surgery alone (*P* < 0.01). In another research, Wang et al^5^ reported their retrospective findings for 81 patients with esophageal squamous cancer; among whom 44 underwent preoperative HFRT with surgery and 47 underwent surgery alone. The patients who underwent preoperative HFRT showed a higher median OS compared with the patients who underwent surgery alone (24.0 months vs 18.0 months).

Although the previous studies of preoperative HFRT followed by surgery indicated that HFRT achieved better outcomes and acceptable toxicity rates compared with surgery alone in patients with resectable esophageal cancer, these trials had certain limitations. They only focused on comparing surgery alone with preoperative HFRT plus surgery, and none compared HFRT with CFRT. Moreover, each of these trials was reported over 20 years ago, and there have undoubtedly been marked improvements in surgical procedures, radiotherapy and chemotherapy over the ensuing 20 years. To the best of our knowledge, no report comparing the efficacies of preoperative HFRT and CFRT in the treatment of esophageal cancer is available.

In the present study, we compared the efficacies of concurrent chemotherapy with preoperative HFRT or CFRT for the treatment of stage II or III esophageal squamous cell cancer. The pCR rate of HFRT (33.3%) was comparable to that of CFRT (35.3%). Pathological downstaging rates were also comparable (*P* = 0.612) at 78.6% for HFRT and 83.8% for CFRT. Kaplan‐Meier analysis confirmed that there were no significant differences in median OS or PFS between the HFRT (40.8 and 32.7 months, respectively; *P* = 0.772) and CFRT (44.8 and 35.4 months, respectively; *P* = 0.785) groups. Therefore, our retrospective data indicated that HFRT is at least non‐inferior to CFRT in terms of the pCR, PFS and OS when treating esophageal cancer.

Given that the oesophagus is a hollow tubular structure, we must consider the potential for HFRT to produce serious side effects, such as esophageal stenosis, haemorrhage, perforation or fistula.^23^ Appropriate total and daily doses must be established for the safe use of HFRT. A phase I/II study of fraction dose escalation indicated that a daily dose of ≤5 Gy is comparatively suitable and tolerated in HFRT for esophageal carcinoma.^18^ A daily dose of 3 Gy is most often used for HFRT in locally advanced esophageal cancer. Ma et al reported relevant findings in 150 patients with thoracic esophageal squamous cell carcinoma (stage T2‐4, N0‐1, M0). Data were prospectively collected for 74 patients who underwent moderate HFRT (MHFRT) with a total dose of 54‐60 Gy in 18‐20 fractions for 3.5‐4 weeks and for 76 patients who underwent CFRT. No significant differences were observed in the incidences of grade 3 or higher acute toxicities (66.3% vs 50.0%, respectively) or of late complications (27.0% vs 22.4%, respectively) between the MHFRT and CFRT arms (*P* > 0.05). A total of six treatment‐related deaths due to esophageal fistulas, pneumonia, cardiotoxicity, or hematological toxicity occurred in the MHFRT arm. Conversely, only two treatment‐related deaths occurred in the CFRT arm. The incidence rates of grade 3 or higher late esophageal complications, including stenosis, fistula or hemorrhage, were similar between the MHFRT and CFRT arms (18.9% vs 21.1%, respectively).^19^

In our study, no significant differences were noted in the occurrence of CRT toxicities or of postoperative complications between the HFRT and CFRT groups, and no deaths occurred within 30 days after surgery. The overall rates of toxicity and treatment‐related deaths observed with HFRT were less compared with previous reports.^4^, ^5^, ^19^ The possible explanation for this is as follows: (a) total preoperative radiotherapy dose in the present study was lower than that used by either Ma et al^19^ or Walsh et al^4^ in their studies, (b) the area of the oesophagus that was subject to the highest radiation dose was resected during surgery, which greatly reduced the potential for radiation‐induced perforations or fistulae and (c) the use of intensity‐modulated radiotherapy in our study allowed the precise delivery of radiation to a target volume that contained only a limited amount of normal tissue, thereby protecting against radiated‐related esophageal, pulmonary, and cardiac injuries.

Under the current payment model, the cost of radiotherapy is based on the number treatments delivered. Although HFRT is reported to be more resource‐efficient and less costly compared with CFRT in patients with breast cancer and prostate cancer,^24^, ^25^, ^26^ to date no study has reported the economic value of preoperative HFRT in esophageal cancer. On comparing the time commitments and costs related to therapy, we found that preoperative HFRT can significantly shorten the durations of both radiotherapy and hospitalization (16.7 and 69.0 days, respectively) compared with CFRT (32.8 and 80.4 days, respectively) by requiring fewer fractions. Moreover, the HFRT schedule required fewer visits to radiation departments, making the approach much more convenient for patients from remote areas. When treatment is completed in a shorter time period, interruptions unrelated to treatment are also reduced, which may improve compliance. Finally, HFRT had significantly lower total and radiotherapy‐related costs compared with CFRT. Overall, our HFRT protocol was advantageous for both the patient and the institute, effectively reducing financial and treatment‐related burdens for both parties.

The limitation of this study was its retrospective nonrandomized design. The reason of applying two different fractionation schemes was because of the physicians' preference. This may bring some selective bias to the selection of patients. Nevertheless, the current results justify randomized prospective clinical trials.

## 5 CONCLUSIONS

We conclude that HFRT has comparable efficacy to CFRT without increasing adverse effect rates or decreasing the pCR, PFS or OS; therefore, it can be recommended as a safe and effective alternative to CFRT for neoadjuvant radiotherapy in patients with esophageal cancer. HFRT requires fewer fractions, can be delivered in a shorter duration and costs less when compared with CFRT. Furthermore, a prospective randomised controlled study with a larger sample is warranted to assess the efficacy of the neoadjuvant HFRT protocol in the treatment of esophageal squamous cell carcinoma to establish HFRT as having a key role in neoadjuvant radiotherapy.

## CONFLICT OF INTEREST

All authors disclose no conflict of interest.
